# Optimal Control Method for Microgrid Distributed Generation Based on Multi-Agent Adaptive Decision-Making

**DOI:** 10.3390/s26102974

**Published:** 2026-05-09

**Authors:** Hao Mai, Qinfang Teng, Xiaojian Wang

**Affiliations:** College of Automation and Electrical Engineering, Lanzhou Jiaotong University, Lanzhou 730050, China; maihao@mail.lzjtu.cn (H.M.); 11230397@stu.lzjtu.edu.cn (X.W.)

**Keywords:** microgrid, distributed generation, multi-agent

## Abstract

In addressing the challenges of strong power generation fluctuation, complex load variation, and dynamic topology changes in microgrid operation, this paper puts forward a proposal for optimal control methodology for distributed generation based on multi-agent adaptive decision-making. The initial conception of the dynamic strategy optimization module is to facilitate online learning and continuous optimization of agent decision-making capabilities. This objective is realized through the construction of a topology correlation matrix, which is based on microgrid operation data. The subsequent generation of preliminary control actions is facilitated by a multi-agent adaptive decision network. Concurrently, a safety optimization model founded upon the barrier Lyapunov function has been developed. The model has been designed to facilitate collaborative correction and safety constraint verification of the preliminary actions, thereby producing final control actions that satisfy global optimization and safe operation requirements. On this basis, an enhanced execution control module is employed, incorporating adaptive ramp rate limitations to calculate the target power reference, achieving high precision and fast tracking of power commands. The findings from both simulation and experimental studies demonstrate that, in complex scenarios such as load fluctuations and topology reconfiguration, the proposed method maintains voltage fluctuations within 1.6 V, achieves frequency recovery within 0.01 s, ensures a rapid power sharing response with a small steady-state error, and improves system operational economy by approximately 8.2% compared to conventional distributed control methods. This enhancement of dynamic adaptability, operational safety, and economic performance of the microgrid is therefore significant.

## 1. Introduction

As an emerging power supply model that integrates various distributed generation resources, the microgrid plays a significant role in promoting the accommodation of clean energy, facilitating the transformation of energy structure, and enhancing power supply reliability [1,2]. In accordance with the carbon peak and carbon neutrality objectives, there has been a consistent increase in the share of renewable energy generation, as represented by photovoltaic and wind power. This has led to the microgrid emerging as a pivotal entity in facilitating the energy transition and attaining low-carbon development. However, with the continuous rise in renewable energy penetration, the operation and control of microgrids face numerous challenges, mainly manifested in the strong volatility and intermittency of power generation, as well as the complexity and uncertainty of load variations, which impose higher demands on system stability and regulation capability. Consequently, within the paradigm of the “dual carbon” objectives, the investigation of optimization control methodologies for microgrids that are adapted to high-proportion renewable energy integration assumes great significance for ensuring safe and efficient system operation and promoting the green transformation of the energy structure.

Insufficient global state awareness and inaccurate dynamic topology modeling in microgrid operation [3] make it difficult for conventional distributed generation control systems to accurately capture the real-time operating characteristics of the system. This, in turn, leads to incomplete decision-making information and delayed responses, thereby affecting control performance and system stability [4]. In order to address the afore-mentioned challenges, artificial intelligence methods have been gradually introduced into the field of microgrid control, leveraging data-driven modeling and intelligent decision-making technologies to enhance the level of automation and intelligence of the system [5]. A series of related studies have been conducted in areas such as state identification, load forecasting and operational optimization [6,7]. Intelligent decision-making mechanisms have been constructed to achieve rapid response and adaptive regulation under complex operating conditions [8,9]. With regard to control strategies, extant research can be principally divided into two categories: centralized optimization and distributed control approaches. Centralized methods realize active and reactive power allocation and operational scheduling of generation units through global optimization models [10,11], offering global coordination capability, but they rely heavily on communication networks and central computing nodes. Rule-based distributed control strategies, on the other hand, achieve power regulation through local information exchange, featuring flexible structures and high reliability, yet they exhibit limitations in multi-objective coordination and adaptability to complex scenarios [12,13].

To further enhance the adaptability and robustness of distributed control, researchers worldwide have carried out extensive studies from aspects such as control architecture, parameter self-tuning, and fault-tolerant mechanisms. Ref. [14] tackled the issue of power fluctuations on tie lines in multi-interconnected microgrids by developing an enhanced interconnection flow controller. With the integration of frequency response control and reactive power management, this controller enables decoupled regulation of active and reactive power, reducing power deviation from ±10% to ±2% during frequency disturbances while cutting the frequency recovery time by over 50%. In addressing the transient response discrepancies among distributed photovoltaic generation systems, Ref. [15] introduced a four-machine equivalent modeling method grounded in active power response characteristics during low-voltage ride-through. By establishing segmented clustering thresholds based on pre-fault steady-state power and voltage sag depth, this approach reduces simulation time by approximately 94.6% without sacrificing model accuracy. Focusing on economic dispatch in multi-microgrid systems, Ref. [16] proposed an improved lyrebird optimization algorithm that leverages Lévy flight and chaotic sine mapping to strengthen both local and global search capabilities. Under reliability-constrained optimization scenarios, this method achieves an active power loss reduction of approximately 1.3% to 2.1%. In terms of microgrid topology, Ref. [17] designed a novel AC/DC hybrid microgrid structure based on silicon-controlled rectifiers and polarity reversal switches. By utilizing the dual functionality of silicon-controlled devices for both rectification and active inversion, the structure supports bidirectional power exchange between the microgrid and the external grid. Combined with a charge–discharge control strategy for energy storage, it achieves annual energy savings of approximately 26,000 kWh in typical daily scenarios across four seasons, effectively reducing equipment costs and control complexity. Regarding reactive power sharing errors caused by feeder impedance mismatch among parallel distributed generation units in islanded microgrids, Ref. [18] presented an improved droop control method based on small AC signal injection. Without requiring communication links or prior knowledge of line impedance parameters, this method injects a small AC signal and extracts its reactive power component to construct a voltage compensation term, thereby achieving accurate active and reactive power sharing while maintaining steady-state voltage harmonic distortion within 1.30%.

In recent years, multi-agent methods have garnered considerable attention in the field of microgrid operation owing to their advantages in distributed decision-making and coordinated control. A coordinated operation strategy for multi-microgrid systems has been developed in Ref. [19] through the training of multi-agent systems, leading to improved overall operational efficiency. A two-layer optimal scheduling method based on multi-agent deep policy gradient has been proposed in Ref. [20], which supports online decision-making and dynamic optimization of equipment regulation, thereby further enhancing the adaptability of control strategies to varying operating conditions. To address the scenario of remote ocean island microgrids lacking ground-based communication infrastructure, Ref. [21] proposed a low-Earth-orbit satellite-assisted over-the-air computation-based federated reinforcement learning scheduling method. In this approach, low-Earth-orbit satellites are innovatively employed as temporary central servers for federated learning, achieving in-channel aggregation of model parameters via over-the-air computation technology. Under the constraints of limited satellite communication bandwidth and computational resources, this method accomplishes economically autonomous operation scheduling for cross-island renewable energy exchange. Nevertheless, despite the improvements in operational efficiency enabled by existing technologies, certain limitations remain. These methods often rely on preset models or fixed parameters [22], making it difficult to adapt to the dynamic and variable operating environment of microgrids. In particular, under complex scenarios such as topological changes and external market disturbances, the control performance tends to be constrained [23]. Moreover, most distributed control methods focus solely on local optimization, failing to effectively coordinate power interactions among multiple units, which can easily lead to safety issues such as voltage violations and frequency fluctuations [24,25]. In addition, the update of existing control strategies largely depends on offline training, limiting the ability to adapt online to changing operating conditions and thereby restricting the long-term optimization capability of the system [26].

To address the aforementioned issues, a distributed generation optimization control method for microgrids based on multi-agent adaptive decision-making is proposed in this paper. The system consists of a data acquisition and topology awareness module, a multi-agent adaptive decision-making module, a distributed safety coordination module, an enhanced execution control module, and a dynamic strategy optimization module. Such a configuration enables rapid generation of practical multi-objective decisions under complex scenarios such as load fluctuations and topology reconfiguration, significantly improving the adaptability and operational economy of the microgrid. The main innovations of this work are summarized as follows:

- A topology recognition algorithm based on an improved Bayesian network is designed to dynamically generate the topology correlation matrix. A weighting mechanism for the state observation vector is introduced, which dynamically assigns weights to each dimension of the multi-source state vector, thereby automatically focusing on critical operational information and effectively alleviating the information overload and decision bias problems inherent in conventional control methods.
- A novel dynamic strategy optimization approach is developed for the adaptive policy network. This approach dynamically adjusts the network structure and activation function parameters according to the information entropy of the state vector. Experience replay samples are screened by information gain, retaining only high-value data, which significantly enhances learning efficiency compared with traditional methods. By incorporating a dynamic clipping coefficient and an adaptive learning rate, online continuous optimization within the control cycle is achieved, overcoming the limitations of conventional offline training.
- The consensus protocol is integrated with the barrier Lyapunov function, enabling global coordination solely through local interactions among neighboring units. This enhances control flexibility and anti-interference capability, allowing global cooperative correction and safety constraint verification without the need for central node intervention.

The structure of this paper is organized as follows. Section 2 presents the construction method of the state observation vector and introduces the design of the adaptive multi-objective decision neural network. Section 3 describes the distributed safety coordination module and the enhanced execution control module, along with the implementation of the safety optimization model based on the barrier Lyapunov function and the enhanced execution control strategy. Section 4 outlines the optimization strategy for the decision neural network. In Section 5, the proposed control method is validated and compared with conventional distributed control approaches. Section 6 concludes the research findings of this paper.

## 2. Microgrid Topology Modeling

**Figure 1:**
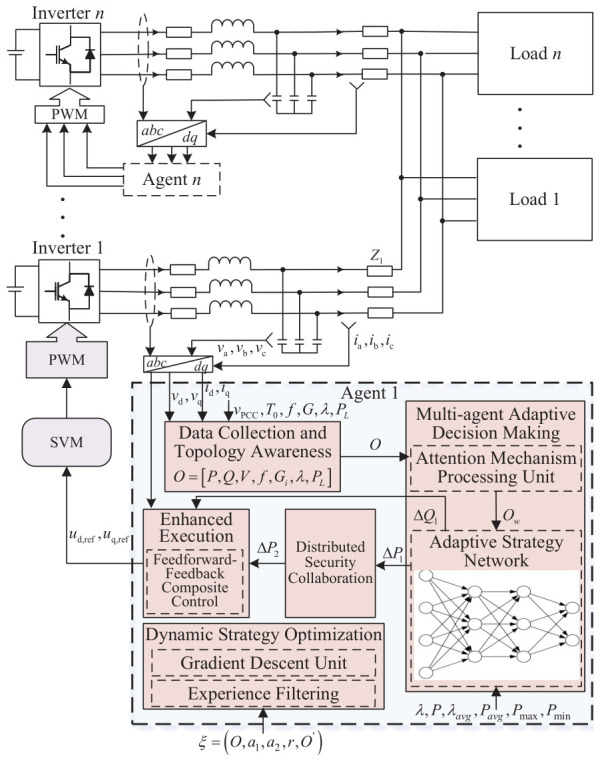
AI-based distributed microgrid control diagram.

The proposed distributed generation optimization control method for microgrids based on multi-agent adaptive decision-making is collaboratively executed by five modules, aiming to achieve intelligent, safe, and coordinated optimization control of distributed generation in microgrids. Each module operates with a control cycle of $T_{c}=50\;\mathrm{ms}$. The system block diagram is illustrated in Figure 1.

(1) Data acquisition and topology awareness module: This module consists of a state sensing unit and a dynamic topology sensing unit. The function of the former is to obtain the measurements required to construct the state observation vector *O*, including instantaneous three-phase voltage values, instantaneous three-phase current values, output active power, reactive power, the root mean square voltage at the point of common coupling of the microgrid, and system frequency. The role of the latter is to detect, in real time, the communication connectivity status and power interaction intensity with adjacent generation units, and to generate the dynamic topology correlation matrix *G* needed for constructing *O* based on an improved Bayesian network [27].
(2) Multi-agent adaptive decision-making module: This module is connected to the data acquisition and topology awareness module. By equipping each generation unit with an independent agent and integrating the state observation vector with an attention mechanism, it generates dynamically adapted multi-objective decisions. This module comprises an attention mechanism processing unit and an adaptive policy network unit. The function of the former is to perform multi-source fusion of state sensing data, the topology correlation matrix, and external environmental parameters, and to dynamically weight each dimension of the state vector through the attention mechanism, thereby constructing the weighted state observation vector $O_{w}$. The role of the latter is to adaptively adjust the structure of the deep neural network and the parameters of the dynamic activation function according to the information entropy of the state vector [28], and to output the preliminary control action $a_{1}$.
(3) Distributed safety coordination module: This module is connected to the multi-agent adaptive decision-making module and consists of a constraint analysis unit for storing the hard constraints of microgrid operation, a coordination verification unit for exchanging decision information with neighboring agents through a consensus protocol and calculating the global coordination error, and a safety correction unit for constructing a safety optimization model based on a barrier Lyapunov function [29] to correct the preliminary decisions.
(4) Enhanced execution control module: This module is connected to the distributed safety coordination module and comprises a reference generation unit and a feedforward–feedback compound control unit. The reference generation unit is used to calculate the target power reference based on the final control action and the current operating power, with an adaptive ramp rate limit incorporated. The feedforward–feedback compound control unit adopts a feedforward–feedback composite control structure, which employs a proportional-resonant regulator to achieve zero steady-state error tracking of the fundamental component, with a feedforward compensation term introduced. This enables the generation of pulse-width modulation drive signals through a space vector modulation algorithm.
(5) Dynamic strategy optimization module: This module is connected to the multi-agent adaptive decision-making module and consists of an experience screening unit and a gradient descent unit. The experience screening unit selects empirical data with high information gain. The gradient descent unit adopts an improved proximal policy optimization algorithm to update network parameters online, thereby achieving continuous evolution and performance optimization of the agent’s decision-making strategy.

### 2.1. Retrieval of the State Observation Vector

As shown in Figure 1, the data acquisition and topology awareness module performs comprehensive sensing and topology modeling of the microgrid operating state, obtaining the state observation vector *O*. *O* encompasses equipment status, topological correlations, and external environmental information, and its expanded expression is given as follows in Equation (1):

(1) $$O=\left[P,Q,V,f,G_{i},\lambda ,P_{L}\right]$$

where *P* and *Q* represent the active power and reactive power of the microgrid system, respectively. These quantities are obtained by performing moving average filtering over 20 power frequency cycles on the instantaneous active power q(t) and instantaneous reactive power p(t). *f* denotes the system frequency obtained by tracking the phase and frequency of the PCC voltage via a phase-locked loop; λ stands for the real-time electricity price; $P_{L}$ represents the load forecast value for the next 15 min. Both λ and $P_{L}$ are acquired through communication with the distribution network dispatch center. The expressions of q(t) and p(t) are given as follows in Equation (2):

(2) $$\left\{\begin{array}{c} q\left(t\right)=\frac{3}{2}\left[v_{q}\left(t\right)i_{d}\left(t\right)+v_{d}\left(t\right)i_{q}\left(t\right)\right]\\ p\left(t\right)=\frac{3}{2}\left[v_{d}\left(t\right)i_{d}\left(t\right)+v_{q}\left(t\right)i_{q}\left(t\right)\right] \end{array}\right.$$

where $v_{d}\left(t\right)$, $v_{q}\left(t\right)$, $i_{d}\left(t\right)$, and $i_{q}\left(t\right)$ are the d-q axis voltages and currents obtained after moving average filtering with a window length of 20 sampling periods, followed by Park transformation of the three-phase instantaneous voltage values $v_{a}\left(t\right)$, $v_{b}\left(t\right)$, and $v_{c}\left(t\right)$ sampled by the voltage sensors and the three-phase instantaneous current values $i_{a}\left(t\right)$, $i_{b}\left(t\right)$, and $i_{c}\left(t\right)$ sampled by the current sensors in the abc axis to the d-q axis.

*V* is the root mean square (RMS) voltage at the point of common coupling (PCC) of the microgrid, and its expression is given as follows in Equation (3):

(3) $$V=\sqrt{\frac{1}{T_{0}}\int_{0}^{T_{0}}v_{\mathrm{PCC}}{\left(t\right)}^{2}\;dt}$$

where $v_{\mathrm{PCC}}\left(t\right)$ is the instantaneous voltage at the PCC; $T_{0}=20\;\mathrm{ms}$ is the power frequency cycle; $G_{i}=\left[g_{i1},g_{i2},\ldots ,g_{iN}\right]$ denotes the *i*-th row of the dynamic topology correlation matrix *G*; *N* is the total number of distributed generation units within the microgrid; and $g_{iN}$ is the element at the *i*-th row and *N*-th column of *G*. A topology correlation calculation model is designed based on an improved Bayesian network, integrating physical distance and power interaction intensity to quantify the topological correlation. $g_{ij}$ denotes the degree of correlation between unit *i* and unit *j*. The element $g_{ij}\in \left[0,1\right]$ at the *i*-th row and *j*-th column of *G* is expressed as in Equation (4):

(4) $$g_{ij}=\frac{\exp\left(-\alpha \cdot d_{ij}\right)\cdot \beta \cdot P_{\mathrm{int},ij}}{\max_{k\neq i}\left(\exp\left(-\alpha \cdot d_{ik}\right)\cdot \beta \cdot P_{\mathrm{int},ik}\right)}$$

where $d_{ij}$ denotes the physical distance between the *i*-th and *j*-th units; α∈[0.008,0.012] represents the distance decay coefficient; β∈[0.7,0.9] is the power weighting coefficient; $P_{\mathrm{int},ij}$ is the power interaction between the *i*-th and *j*-th units; and $P_{\mathrm{int},ik}$ is the power interaction between the *i*-th and *k*-th units. The dynamic topology sensing unit establishes communication with neighboring generation units via a CAN bus at a period of 10 ms, with each unit periodically broadcasting its own device ID, operating status, and real-time output power. When communication between two units is interrupted for more than three communication cycles or when $g_{ij}<0.1$, $g_{ij}=0$, indicating the absence of a direct topological association; correspondingly, $g_{ij}=1$ indicates the strongest topological association. The communication efficiency issue has been fully addressed by adopting a selective communication mechanism based on the degree of topological correlation, rather than the conventional fully connected broadcast mode, thereby fundamentally reducing communication overhead. Each distributed generation unit periodically broadcasts only three core data items: device ID (1 byte), operating status (1 byte), and real-time output power (4 bytes). The total length of a single message is only 6 bytes, which is less than the 8-byte upper limit of the standard CAN bus data frame. Furthermore, decision information is exchanged only with neighboring units whose topological correlation degree satisfies $g_{ij}\geq 0.3$, rather than with all units in the system. As the system scale expands, the number of communication neighbors for each unit remains relatively stable, and the communication overhead does not increase linearly with system size. Meanwhile, the total length of a complete CAN bus frame can be calculated. Considering the frame header, control bits, CRC, ACK, etc., which total approximately 17 bytes, together with the 6-byte message length, the total frame length is approximately 23 bytes, i.e., 184 bits. With a communication period of 10 ms, the bandwidth occupancy per unit is only 18.4 kbps. Even if the system is expanded to 20 distributed generation units, the total bandwidth occupancy is less than 400 kbps, which is below the theoretical maximum bandwidth of the CAN bus and meets real-time requirements.

Upon completion of the above steps, full-dimensional data acquisition and preprocessing are accomplished. The dimension of the state observation vector *O* is (N+6), covering equipment status, topological associations, and external environmental information.

### 2.2. Weighted State Observation Vector

Subsequently, to enable the multi-agent decision-making system to automatically focus on key influencing factors and to avoid information overload and decision bias, an attention mechanism is introduced to dynamically weight the state observation vector *O*. The weighted state observation vector, denoted as $O_{w}$, is constructed by the attention mechanism processing unit and expressed as follows in Equation (5):

(5) $$O_{w}=W\cdot O$$

where, $W\in R^{\left(N+6)\times (N+6\right)}$ is the attention weight matrix, whose diagonal elements $w_{k}$ represent the attention weights for the corresponding state dimensions. The calculation method is given by Equation (6):

(6) $$w_{k}=\frac{\sigma (s_{k})}{\sum_{k=1}^{N+6}\sigma (s_{k})}$$

where $s_{k}\in \left[0,1\right]$ is the normalized value of the state dimension, and σ(·) denotes the Softmax activation function.

Through such weighting, the decision-making system is enabled to adaptively focus on the critical factors of the microgrid, thereby overcoming the limited adaptability of fixed-weight models under complex operating conditions.

### 2.3. Adaptive Multi-Objective Decision-Making Neural Network

As illustrated in Figure 1 the designed objective decision neural network takes $O_{w}$ as its input and outputs the active power adjustment $\Delta P_{1}$ and the auxiliary reactive power adjustment $\Delta Q_{1}$. The network design is detailed as follows:

(1) Network activation function: A dynamic Exponential Linear Unit (ELU) function is adopted, which adjusts the attenuation characteristics in the negative region according to the complexity of operating conditions:

(7) $$\sigma_{d}\left(x\right)=\left\{\begin{array}{cc} x & x\geq 0\\ \alpha_{d}\left(\exp(x)-1\right) & x<0 \end{array}\right.$$

In the expression, *x* is the input to the dynamic ELU function, i.e., $O_{w}$; and $\alpha_{d}$ is the attenuation coefficient in the negative region of the ELU function.

The adjustment method for $\alpha_{d}$ is as follows:

(8) $$\alpha_{d}=0.5+0.3\cdot e^{-\frac{H(O_{w})}{3.2}}$$

where $H\left(\cdot \right)$ denotes the information entropy.

The expression of the output $h_{i}$ of the *i*-th hidden layer of the neural network is given as

(9) $$h_{i}=\left\{\begin{array}{c} \sigma_{d}\left(W_{i}O_{w}+b_{i}\right),\;i=1\\ \sigma_{d}\left(W_{i}h_{i-1}+b_{i}\right),\;i\neq 1 \end{array}\right.$$

The output $a_{\mathrm{nn}}$ of the output layer of the neural network is expressed as:

(10) $$a_{\mathrm{nn}}=\left[\begin{array}{c} \Delta P_{1}\\ \Delta Q_{1} \end{array}\right]=W_{L}h_{L-1}+b_{L}$$

where *L* is the number of hidden layers. When expressed in the form of a policy function, it follows

(11) $$a_{\mathrm{nn}}=\pi_{\varphi }\left(O_{w}\right)$$

where $\varphi =\left\{W_{1},\;b_{1},\;\ldots ,\;W_{L},\;b_{L},\;\alpha_{d}\right\}$ represents all learnable parameters of the network. The specific learning method is introduced in Section 4.

(2) Network output: The output consists of the preliminary control actions that balance economic benefits and system stability, namely the active power adjustment $\Delta P_{1}$ and the auxiliary reactive power adjustment $\Delta Q_{1}$. The preliminary control action is denoted as $a_{1}=\Delta P_{1}$, with its value range is set to $\left[\Delta P_{\min},\Delta P_{\max}\right]$, where $\Delta P_{\min}$ and $\Delta P_{\max}$ are the lower and upper bounds of $a_{1}$ determined based on actual operating conditions. $\Delta Q_{1}$ is employed for directly adjusting the reactive power of the system; $\Delta P_{1}$ is processed by the distributed safety coordination module to obtain the final active power adjustment action.

(3) Network loss function: To achieve multi-objecive optimization, a multi-objective loss function *L* is introduced, expressed as in Equation (12):

(12) $$L=w_{1}L_{e}+w_{2}L_{s}+w_{3}L_{\mathrm{sf}}$$

where, $w_{1}$, $w_{2}$, and $w_{3}$ are dynamic weight coefficients, whose sum equals 1. The specific values are dynamically adjusted by a fuzzy logic controller. The fuzzy logic for $w_{1}$, $w_{2}$, and $w_{3}$ is defined as follows [30]: when system fluctuations are significant, $w_{2}\in \left[0.55,0.65\right]$; during peak electricity price periods, $w_{1}\in \left[0.45,0.55\right]$; when power approaches its limits, $w_{3}\in \left[0.45,0.55\right]$. This ensures that the decision-making process balances multiple objectives. $L_{e}$ is the economic loss term, used to quantify the deviation in economic benefit; $L_{s}$ is the stability loss term, which reflects the operational stability of the system; and $L_{\mathrm{sf}}$ is the safety loss term, which ensures that power output remains within safe limits. The expressions for $L_{e}$, $L_{s}$, and $L_{\mathrm{sf}}$ are given respectively as in Equation (13):

(13) $$\left\{\begin{array}{c} L_{e}={\left(\lambda P-\lambda_{\mathrm{avg}}P_{\mathrm{avg}}\right)}^{2}\\ L_{s}={\left(f-f_{\mathrm{nom}}\right)}^{2}+{\left(V-V_{\mathrm{nom}}\right)}^{2}\\ L_{\mathrm{sf}}=\max{\left(0,P-P_{\max}\right)}^{2}+\max{\left(0,P_{\min}-P\right)}^{2} \end{array}\right.$$

where $\lambda_{\mathrm{avg}}$ represents the average electricity price over the past hour, $P_{\mathrm{avg}}$ is the average power over the past hour, and $P_{\max}$ and $P_{\min}$ denote the maximum and minimum allowable active power, respectively.

## 3. Distributed Safety Coordination and Enhanced Execution Control Module

### 3.1. Distributed Coordination and Safety Correction

As shown in Figure 1, this step is performed by the distributed safety coordination module, which constructs an architecture for deep integration of distributed coordination and safety constraints, enabling global optimization without the need for central node intervention. First, the hard constraint parameters are read by the constraint analysis unit:

(1) Constraints on $P_{\min}$: For photovoltaic units are given as in Equation (14):

(14) $$P_{\min}\in \left[0.08P_{\max},0.12P_{\max}\right]$$

For energy storage units are given as in Equation (15):

(15) $$P_{\min}=-P_{\max}$$

(2) Constraints on *V* and *f* are given as in Equation (16):

(16) $$\left\{\begin{array}{c} V\in \left[0.95V_{\mathrm{nom}},1.05V_{\mathrm{nom}}\right],\;V_{\mathrm{nom}}=380\;V\\ f\in [49.5,50.5] \end{array}\right.$$

where $V_{\mathrm{nom}}$ denotes the rated voltage.

(3) Distributed coordination constraints: These are obtained through distributed power flow calculations without requiring central node allocation, as expressed below in Equation (17):

(17) $$\left\{\begin{array}{c} \sum_{i=1}^{N}\Delta P_{1i}=\Delta P_{\mathrm{req}}\\ \Delta P_{\mathrm{req}}=P_{L}-\sum_{i=1}^{N}P_{ci} \end{array}\right.$$

where $\Delta P_{1i}$ is the preliminary action of the *i*-th agent, and each agent sends its own $\Delta P_{1}$ while receiving $\Delta P_{1}$ from neighboring units; $P_{ci}$ is the current operating power of the *i*-th agent; and $\Delta P_{\mathrm{req}}$ is the total system power adjustment requirement.

The coordination verification unit exchanges decision information with neighboring units satisfying $g_{ij}\geq 0.3$ via a consensus protocol, thereby selecting highly correlated units for coordination and reducing invalid communication overhead and network latency. The global coordination error $e_{c}$ is calculated as in Equation (18):

(18) $$e_{c}=\left|\sum_{i=1}^{N}\Delta P_{1i}-\Delta P_{\mathrm{req}}\right|$$

When $e_{c}\leq 0.05P_{L}$, no correction is required; otherwise, the coordination correction amount is allocated based on the topological correlation degree $g_{ij}$ as follows in Equation (19):

(19) $$\Delta P_{c}=\frac{g_{i,\mathrm{sum}}}{\sum_{i=1}^{N}g_{i,\mathrm{sum}}}\cdot \left(-\frac{e_{c}}{N}\right)$$

$g_{i,\mathrm{sum}}=\sum_{i=1}^{N}g_{ij}$ is the sum of topological correlations for the *i*-th unit. After coordination correction, $\Delta {P^{\prime }}_{2}=\Delta P_{1}+\Delta P_{c}$ is obtained. This correction approach achieves global coordination through local interactions between neighboring units without requiring central coordination, thereby enhancing control flexibility and anti-interference capability.

The safety correction unit constructs a safety optimization model based on a barrier Lyapunov function. The voltage safety barrier function $B_{V}$, frequency safety barrier function $B_{f}$, and power safety barrier function $B_{P}$ are defined respectively as in Equation (20):

(20) $$\left\{\begin{array}{c} B_{V}=\left(V-V_{\min}\right)\left(V_{\max}-V\right)\\ B_{f}=\left(f-f_{\min}\right)\left(f_{\max}-f\right)\\ B_{P}=\left(P-P_{\min}\right)\left(P_{\max}-P\right) \end{array}\right.$$

where $V_{\min}$ and $V_{\max}$ represent the lower and upper limits of the grid voltage, respectively, and $f_{\min}$ and $f_{\max}$ denote the lower and upper limits of the grid frequency, respectively.

The safety constraints are expressed as in Equation (21):

(21) $$\left\{\begin{array}{c} {\dot{B}}_{V}+k_{V}B_{V}\geq 0\\ {\dot{B}}_{f}+k_{f}B_{f}\geq 0\\ {\dot{B}}_{P}+k_{P}B_{P}\geq 0 \end{array}\right.$$

where $k_{V}\in \left[0.7,0.9\right]$, $k_{f}\in \left[0.9,1.1\right]$, and $k_{P}\in \left[0.5,0.7\right]$ are the decay coefficients of the respective functions; and ${\dot{B}}_{V}$, ${\dot{B}}_{f}$, and ${\dot{B}}_{P}$ are the first-order time derivatives of the corresponding barrier functions, expressed as in Equation (22):

(22) $$\left\{\begin{array}{c} {\dot{B}}_{V}=\frac{dB_{V}}{dV}\cdot \frac{dV}{dt}\\ {\dot{B}}_{f}=\frac{dB_{f}}{df}\cdot \frac{df}{dt}\\ {\dot{B}}_{P}=\frac{dB_{P}}{dP}\cdot \frac{dP}{dt} \end{array}\right.$$

Through system identification, the coupling relationships between power adjustments and voltage and frequency are obtained as in Equation (23):

(23) $$\left\{\begin{array}{c} \frac{dV}{dt}=k_{\mathrm{PV}}\Delta {P^{\prime }}_{2}\\ \frac{df}{dt}=k_{\mathrm{PF}}\Delta {P^{\prime }}_{2} \end{array}\right.$$

where $k_{\mathrm{PV}}\in \left[0.015,0.025\right]$ and $k_{\mathrm{PF}}\in \left[0.008,0.012\right]$. $k_{\mathrm{PV}}$ and $k_{\mathrm{PF}}$ denote the power–voltage coupling coefficient and the power–frequency coupling coefficient, respectively. $\Delta {P^{\prime }}_{2}$ denotes the candidate final power adjustment.

Finally, the final active power adjustment $\Delta P_{2}$ is determined. If $\Delta {P^{\prime }}_{2}$ satisfies the constraints in Equation (21), it is directly assigned as in Equation (24):

(24) $$\Delta P_{2}=\Delta {P^{\prime }}_{2}$$

If $\Delta {P^{\prime }}_{2}$ does not satisfy the constraints in Equation (21), a safety optimization problem is formulated to satisfy the safety constraints while preserving the coordination correction intent as in Equation (25):

(25) $$\left\{\begin{array}{c} \min_{\Delta P_{2}}\;{\left(\Delta P_{2}-\Delta {P^{\prime }}_{2}\right)}^{2}\\ \begin{array}{cc} s.t. & {\dot{B}}_{V}+k_{V}B_{V}\geq 0\\ & {\dot{B}}_{f}+k_{f}B_{f}\geq 0\\ & {\dot{B}}_{P}+k_{P}B_{P}\geq 0 \end{array} \end{array}\right.$$

This convex quadratic programming problem is solved using the interior point method, with a solution time of less than 10 ms. The resulting $\Delta P_{2}$ satisfies both coordination and safety requirements, effectively avoiding local optima and safety risks. Denoting $\Delta P_{2}$ as the final action $a_{2}$ it follows that Equation (26) holds:

(26) $$a_{2}=\Delta P_{2}$$

The invariance proof of the safety constraints and the global asymptotic stability analysis of the closed-loop system are presented below. It is thereby demonstrated that the proposed control method can always confine the system state within the predefined safe region from any initial safe state, while ensuring global convergence to the desired equilibrium point.

First, the global safe region D for microgrid operation is defined as the set of all states satisfying the hard constraints:

(27) $$D=\left\{x\in R^{3}\left|V\in \left[V_{\min},V_{\max}\right],f\in \left[f_{\min},f_{\max}\right],P\in \left[P_{\min},P_{\max}\right]\right.\right\}$$

**Theorem** **1.** *If the initial system state x(0)∈int(D) and $\Delta P_{2}$ satisfies the barrier constraint conditions (i.e., Equation (17) in the original manuscript), then for any t≥0 it holds that x∈int(D); that is, the system state never leaves the boundary of the safe region D.*

**Proof.**  Taking the voltage safety constraint as an example, multiply both sides of the inequality ${\dot{B}}_{V}+k_{V}B_{V}\geq 0$ by $e^{k_{V}t}$ to obtain

(28) $$\frac{d}{dt}\left(B_{V}e^{k_{V}t}\right)\geq 0\Rightarrow B_{V}\left(t\right)\geq B_{V}\left(0\right)e^{-k_{V}t}$$

Since the initial state x(0)∈int(D), we have $B_{v}\left(0\right)>0$. Moreover, because $k_{v}>0$, it follows that for any t≥0 $B_{v}\left(0\right)>0$. Combined with the vanishing property of the barrier function at the boundary, it can be concluded that the voltage $V\left(t\right)$ never reaches $V_{\min}$ or $V_{\max}$, i.e., $V\left(t\right)\in \left(V_{\min},V_{\max}\right)$. By the same reasoning, it can be proved that $f\left(t\right)\in \left(f_{\min},f_{\max}\right)$ and $P\left(t\right)\in \left(P_{\min},P_{\max}\right)$. Thus, the system state always remains inside the safe region *D*, and the safety constraints are globally invariant. □

Next, the global asymptotic stability of the closed-loop system is analyzed. The closed-loop state vector is defined as

(29) $$x={\left[\tilde{V},\tilde{f},\tilde{P}\right]}^{T}$$

where $\tilde{V}=V-V_{ref}$, $\tilde{f}=f-f_{ref}$, and $\tilde{P}=P-P_{ref}$ are the errors of voltage, frequency, and active power with respect to the desired equilibrium point $x^{*}={\left[380\;V,50\;\mathrm{Hz},P_{\mathrm{ref}}\right]}^{T}$.

A global composite Lyapunov function is constructed as

(30) $$V_{\mathrm{total}}\left(x\right)=\frac{1}{2}B_{V}\left(\tilde{V}\right)+\frac{1}{2}B_{f}\left(\tilde{f}\right)+\frac{1}{2}B_{P}\left(\tilde{P}\right)$$

From Equation (4) (in the original manuscript), it can be seen that $V_{\mathrm{total}}\left(x\right)$ is positive definite in $\mathrm{int}\left(D\right)$, and

(31) $$\left\{\begin{array}{c} \lim_{x\to \partial D}V_{\mathrm{total}}\left(x\right)=0\\ \lim_{x\to x*}V_{\mathrm{total}}\left(x\right)=V_{\mathrm{total}}^{*} \end{array}\right.$$

where $V_{total}^{*}$ is a finite positive value.

Differentiating $V_{total}\left(x\right)$ along the closed-loop system trajectory yields

(32) $${\dot{V}}_{\mathrm{total}}\left(x\right)=\frac{1}{2}{\dot{B}}_{V}+\frac{1}{2}{\dot{B}}_{f}+\frac{1}{2}{\dot{B}}_{P}$$

Combined with the barrier constraint conditions of Equation (17) in the original manuscript, we obtain

(33) $${\dot{V}}_{\mathrm{total}}\left(x\right)\leq -\frac{1}{2}k_{V}B_{V}-\frac{1}{2}k_{f}B_{f}-\frac{1}{2}k_{P}B_{P}=-KV_{\mathrm{total}}$$

where $K=\min\left\{k_{v},k_{f},k_{p}\right\}$.

According to Lyapunov stability theory, since $V_{total}\left(x\right)$ is positive definite and ${\dot{V}}_{total}\left(x\right)$ is negative definite, the closed-loop system is globally asymptotically stable at the equilibrium point. Furthermore, solving the differential inequality gives

(34) $$V_{\mathrm{total}}\left(t\right)\leq V_{\mathrm{total}}\left(0\right)e^{-Kt}$$

It indicates that the system state errors converge to zero at an exponential rate.

Finally, the global stability under distributed coordination is discussed. For the multi-agent distributed system, each agent exchanges information only with its neighbors whose topological correlation satisfies $g_{ij}\geq 0.3$, and global power coordination is achieved through a consensus protocol. This is because (1) the topology correlation matrix *G* is symmetric and positive semi-definite, and any two agents are connected by a communication path during normal operation of the microgrid; (2) the local control law of each agent satisfies the Lyapunov stability conditions described above; and (3) the global coordination error $e_{c}\leq 0.05P_{L}$ does not trigger correction, ensuring continuity of the control action. Consequently, the composite Lyapunov function of the entire distributed system is the sum of the local Lyapunov functions of the agents, and its derivative remains negative definite. Thus, global asymptotic stability under distributed coordination is established without the need for central node intervention.

Moreover, the convex quadratic programming problem of Equation (21) in the original manuscript is solved using the interior point method. Since (1) the objective function ${\left(\Delta P_{2}-\Delta P_{2}^{\prime }\right)}^{2}$ is strictly convex; (2) the constraints (i.e., Equation (17) in the original manuscript) are all linear inequality constraints; and (3) the initial state lies inside the safe region and $\Delta P_{2}=0$ is always a feasible solution, this convex quadratic programming problem has a unique optimal solution, guaranteeing the feasibility of the control action.

### 3.2. Enhanced Execution Control Module

As shown in Figure 1, this step is performed by the enhanced execution control module, which adopts a feedforward–feedback compound control structure and a dynamic reference generation mechanism to improve power tracking accuracy and anti-interference capability.

First, the reference generation unit calculates the power reference $P_{\mathrm{ref}}$ and is given as in Equation (35):

(35) $$P_{\mathrm{ref}}=P_{c}+\Delta P_{2}$$

A ramp rate limit is concurrently applied to mitigate the impact of abrupt power changes:

(36) $$P_{\mathrm{ref}}=\left\{\begin{array}{cc} P_{c}+\Delta P_{r} & \Delta P_{2}\Delta P_{r}\\ P_{c}+\Delta P_{2} & \left|\Delta P_{2}\right|\leq \Delta P_{r}\\ P_{c}-\Delta P_{r} & \Delta P_{2}-\Delta P_{r} \end{array}\right.$$

where $\Delta P_{r}=0.1\frac{P_{\max}\;}{T_{c}}$ is the maximum allowable power change rate per unit time, adaptively set according to the power regulation capability of the generation unit to ensure a smooth power transition; and $T_{c}$ is the control cycle. Subsequently, the compound control unit adopts a feedforward–feedback compound control structure, where the feedback channel employs a proportional-resonant (PR) regulator. The transfer function $G_{\mathrm{PR}}\left(s\right)$ of the controller is given by Equation (37):

(37) $$G_{\mathrm{PR}}\left(s\right)=k_{p}+\sum_{n\in \left\{1,3,5\right\}}\frac{k_{r}s}{s^{2}+{\left(2\;\;\;\pi \;\;\;n\times f_{\mathrm{nom}}\right)}^{2}}$$

where $k_{p}\in \left[0.7,0.9\right]$ is the proportional coefficient, and $k_{r}\in \left[18,22\right]$ is the resonant coefficient.

To achieve zero steady-state error tracking of the fundamental component and suppress dominant harmonics, grid voltage feedforward $v_{ff}$ and load disturbance feedforward $i_{ff}$ are introduced into the feedforward channel, expressed respectively as in Equation (38):

(38) $$\left\{\begin{array}{c} v_{ff}={\hat{v}}_{\mathrm{grid}}\left(t\right)\\ i_{ff}={\hat{i}}_{L}\left(t\right)\cdot Z_{\mathrm{eq}} \end{array}\right.$$

where ${\hat{v}}_{\mathrm{grid}}\left(t\right)$ is the observed grid voltage, ${\hat{i}}_{L}\left(t\right)$ is the observed load current, and $Z_{\mathrm{eq}}\in \left[0.4+j0.2,0.6+j0.4\right]\Omega$ is the equivalent system impedance used to preemptively counteract external disturbances.

Finally, the output of the PR regulator is superimposed with the feedforward compensation terms to generate the d-q axis voltage references $v_{d,\mathrm{ref}}$ and $v_{q,\mathrm{ref}}$. The six PWM drive signals are generated using the space vector modulation (SVM) algorithm, which drives the IGBT switches to achieve high-precision tracking of the power reference $P_{\mathrm{ref}}$. The durations of adjacent active vectors and the zero vector are calculated as follows in Equation (39):

(39) $$\left\{\begin{array}{c} t_{1}=\sqrt{3}V_{\mathrm{ref}}T_{s}\sin\left(\frac{\;\;\;\pi \;\;\;}{3}-\theta \right)/U_{\mathrm{dc}}\;\\ t_{2}=\sqrt{3}V_{\mathrm{ref}}T_{s}\sin\theta /U_{\mathrm{dc}}\;\\ t_{0}=T_{s}-t_{1}-t_{2} \end{array}\right.$$

where $V_{\mathrm{ref}}=\sqrt{v_{d,\mathrm{ref}}^{2}+v_{q,\mathrm{ref}}^{2}}$; $T_{s}=100\;\mu s$ is the switching period; θ is the phase angle of the reference voltage vector; and $U_{\mathrm{dc}}=700\;V$ is the DC bus voltage.

## 4. Optimization Strategy for the Decision Neural Network

The optimization strategy for the decision neural network is executed by the dynamic strategy optimization module, which establishes a mechanism for effective experience screening and dynamic parameter adjustment to enable online evolution of the decision-making strategy.

First, the experience screening unit collects experience data ξ, which specifically includes Equation (40):

(40) $$\xi =(O,a_{1},a_{2},r,O^{\prime })$$

where $O^{\prime }$ represents the state at the next time step; r∈[0,3] is the reward value, which serves to comprehensively evaluate economic benefits, system stability, and operational safety. A larger value of *r* indicates better control performance, and the multi-dimensional reward guides the policy toward optimal comprehensive performance. Its expression is given by Equation (41):

(41) $$r=r_{e}+r_{s}+r_{\mathrm{sf}}\;r\in \left[0,3\right]$$

where $r_{e}$ is the economic reward, $r_{s}$ is the stability reward, and $r_{\mathrm{sf}}$ is the safety reward. Their respective expressions are Equation (42):

(42) $$\left\{\begin{array}{c} r_{e}=1-\frac{\left|\lambda P-\lambda_{\mathrm{peak}}P_{\max}\right|}{\lambda_{\mathrm{peak}}P_{\max}}\\ r_{s}=1-\frac{\left|f-f_{\mathrm{nom}}\right|}{1.0}-\frac{\left|V-V_{\mathrm{nom}}\right|}{38}\\ r_{\mathrm{sf}}=1-\frac{\max\left(0,P-P_{\max}\right)+\max\left(0,P_{\min}-P\right)}{P_{\max}-P_{\min}} \end{array}\right.$$

where $\lambda_{\mathrm{peak}}$ denotes the peak electricity price of the day.

Second, to address the inefficiency caused by redundancy in the experience buffer, an information gain screening mechanism is adopted. Information entropy H(·) is defined as a quantitative measure of the uncertainty of random variables, and the information gain threshold can be adjusted according to optimization accuracy. The information gain $G_{\mathrm{info}}$ for each experience is calculated as in Equation (43):

(43) $$G_{\mathrm{info}}=H\left(O\right)-H\left(O\left|O^{\prime }\right.\right)$$

where $H(O\left|O^{\prime }\right.)$ is the conditional information entropy of the current state *O* given the next state $O^{\prime }$. A ring buffer with a capacity of $10^{5}$ entries is set as the experience replay buffer. Only when $G_{\mathrm{info}}\geq 0.2$ is the experience ξ considered to contain valid new information and stored in the buffer. When the buffer reaches its capacity, the experience with the smallest information gain is removed, ensuring that only high-value experiences are retained, thereby improving the efficiency and effectiveness of policy optimization.

The gradient descent unit adopts an improved proximal policy optimization (PPO) algorithm [31] to update the parameter set φ comprising the weights and biases of the policy network. This algorithm employs a clipping mechanism to limit the magnitude of policy updates, thereby avoiding training instability caused by excessively rapid parameter changes. On this basis, a dynamic parameter adjustment mechanism is introduced to further enhance convergence performance and adaptability. A mini-batch of 256 valid experiences is randomly sampled from the experience buffer, and the generalized advantage estimation (GAE) method is used to compute the advantage function estimate $\hat{A}$, improving the accuracy of advantage estimation. The expression is given as in Equation (44):

(44) $$\left\{\begin{array}{c} {\hat{A}}_{t}=\sum_{k=0}^{T-1}{\left(\gamma^{\prime }\cdot \lambda_{\mathrm{gae}}\right)}^{k}\delta_{t+k}\\ \delta_{t}=r_{t}+\gamma^{\prime }V_{\psi^{\prime }}\left(O_{t+1}\right)-V_{\psi }\left(O_{t}\right) \end{array}\right.$$

where *t* is the time step index of the experience data; *k* is the temporal step offset used to accumulate temporal-difference errors at different offsets after the current time step; *T* is the length of the sampled sequence; $\gamma^{\prime }\in \left[0.98,0.99\right]$ is the discount factor, balancing the importance of current and future rewards; $\lambda_{\mathrm{gae}}\in \left[0.94,0.96\right]$ is the GAE parameter, balancing bias and variance in advantage estimation; $\delta_{t}$ is the temporal-difference error; $r_{t}$ is the reward value corresponding to the *t*-th time step (i.e., the *t*-th control cycle), consistent in meaning with the reward *r* for a single control cycle, representing its temporal representation at a specific time step; $V_{\psi }\left(\cdot \right)$ is the value network used to estimate the state value; ψ is the parameter set of the value network; $V_{\psi^{\prime }}\left(\cdot \right)$ is the target value network; and $\psi^{\prime }$ is the parameter set of the target value network, which is synchronized with $\psi^{\prime }$ = ψ every 10 policy updates to avoid value estimation bias.

Meanwhile, the dynamic clipping parameter ι(t) and adaptive learning rate η(t) are introduced, which are dynamically adjusted according to the training step *t* to balance policy exploration and convergence stability. Their expressions are given as in Equation (45):

(45) $$\left\{\begin{array}{c} \iota \left(t\right)=0.2-0.1\cdot \exp\left(-\frac{t}{10^{4}}\right)\\ \eta \left(t\right)=0.001\cdot \frac{1}{\sqrt{1+0.001t}} \end{array}\right.$$

As shown in Equation (33), in the early stage of training when *t* is small, ι(t) approaches 0.2, supporting rapid policy exploration to adapt to diverse operating conditions. In the later stage of training when *t* becomes large, ι(t) approaches 0.1, limiting the magnitude of policy updates to ensure convergence stability. The adaptive learning rate η(t) decays gradually with the number of training steps, preventing parameter oscillations caused by an excessively large learning rate in the later stage, thereby improving convergence accuracy.

The loss function of the improved PPO algorithm is designed as follows, incorporating an entropy regularization term to encourage policy exploration and avoid falling into local optima, as shown in Equation (46):

(46) $$\begin{array}{cc} L\left(\varphi \right)=E_{\xi ∼D} & [\min\left(r\left(\varphi \right)\hat{A},\;\mathrm{clip}\left(r\left(\varphi \right),1-\iota \left(t\right),1+\iota \left(t\right)\right)\hat{A}\right)]-\beta^{\prime }H\left(\pi_{\varphi }\left(a_{1}|O\right)\right) \end{array}$$

where *D* denotes the experience replay buffer; $E_{\xi ∼D}\left[\cdot \right]$ denotes the expectation over the data in the buffer; $r\left(\varphi \right)=\frac{\pi_{\varphi }\left(a_{1}|O\right)}{\pi_{\varphi_{\mathrm{old}}}\left(a_{1}|O\right)}$ is the policy probability ratio; $\pi_{\varphi }\left(a_{1}|O\right)$ is the probability density of outputting action $a_{1}$ under the current policy with parameters φ given state *O*; $\pi_{\varphi_{\mathrm{old}}}\left(a_{1}|O\right)$ is the probability density under the old policy with parameters $\varphi_{\mathrm{old}}$ before the update; clip(·) is the clipping function that limits the policy probability ratio to the interval [1−ι(t),1+ι(t)]; $\beta^{\prime }$ is the entropy regularization coefficient; and $H\left(\pi_{\varphi }\left(a_{1}|O\right)\right)$ is the entropy of the current policy, which increases the randomness of the policy through the entropy term, encouraging exploration toward better decisions.

The Adam optimizer is employed to minimize the loss function and update the policy network parameters φ. The update formula is given in Equation (47):

(47) $$\varphi =\varphi -\eta \left(t\right)\cdot \nabla_{\varphi }L\left(\varphi \right)$$

where $\nabla_{\varphi }L\left(\varphi \right)$ is the gradient of the loss function L(φ) with respect to the parameters φ. Each parameter update involves 10 iterations to ensure adequate optimization, and a policy update is performed every 50 control cycles to balance optimization effectiveness and real-time performance. Through this dynamic policy optimization mechanism, the adaptive policy network is able to continuously learn and evolve in response to dynamic changes in microgrid operating conditions, consistently maintaining optimal decision-making performance in complex and varying operational scenarios, thereby significantly enhancing the adaptability and robustness of the control system.The definitions of all variable symbols are presented in Table 1.

## 5. Validation of the Control Method and Comparison of Results

**Figure 2:**
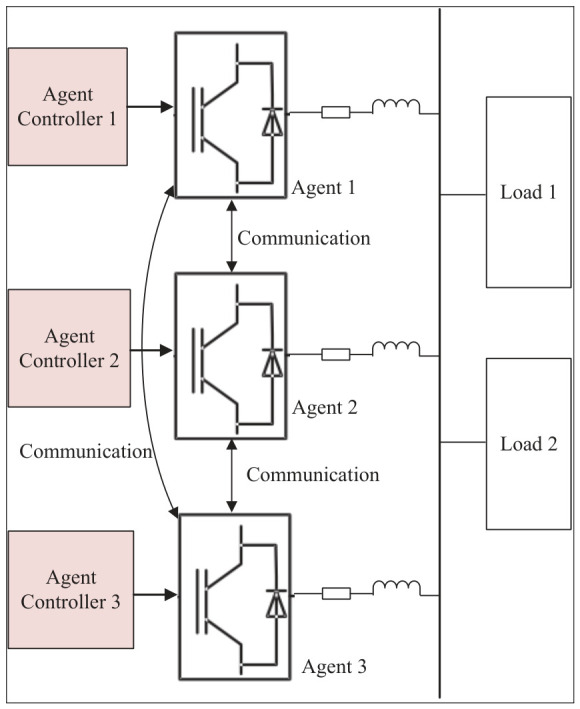
Distributed energy system.

To verify the effectiveness of the proposed algorithm, a simulation experiment based on the MATLAB/Simulink R2022a platform was designed to emulate a distributed energy system consisting of three agents, as shown in Figure 2. This system comprises three generation units and two loads, with each generation unit serving as an agent connected to its neighboring agents for mutual communication. For the first two scenarios, an additional microgrid system adopting droop control under identical conditions is established for comparison. All simulation parameters are listed in Table 2.

Parameter tuning principle:

Specifically, the threshold for topology correlation degree is determined based on statistical analysis of CAN bus communication reliability and power coupling characteristics between units. When $g_{ij}<0.1$, the power interaction between units is very small, and the probability of no data exchange for three consecutive communication cycles is extremely high. Therefore, it is judged that there is no direct topological correlation, and the unit can be excluded from coordination to reduce communication overhead.

The threshold of 0.2 for the information gain $G_{\mathrm{info}}$ is determined as the optimal value through comparative simulations with a step size of 0.05 in the interval [0.1,0.5]. When the threshold is too low, the training speed is slow and the policy tends to oscillate; when the threshold is too high, valuable experience is significantly lost, and convergence stability decreases. At 0.2, the best balance among experience utilization, convergence speed, and control robustness is achieved.

For the controller gains and other parameters, we did indeed use a simulation trial-and-error approach. In the design process, we first gave the initial range of parameters based on the small-signal model of the microgrid, inverter bandwidth requirements, and system stability margins. Then we gradually optimized through simulation tests, finally determining a set of optimal parameters that balance response speed, steady-state accuracy, and robustness. Although this process has a certain trial-and-error nature, simulations show that when the parameters fluctuate within a reasonable range, key indicators such as voltage, frequency, and power tracking still meet microgrid operation specifications, without instability, oscillation, or significant performance degradation.

The fuzzy weight rules are formulated based on the safety priority of microgrid operation and multi-objective optimization preferences: when the system experiences large fluctuations, stability is prioritized; during peak electricity price periods, economy is prioritized; when power approaches limits, safety constraints are prioritized. The weight intervals are determined through simulation comparisons to ensure a balance among economy, stability, and safety under different operating conditions.

The scheduling arrangement of the PPO algorithm is determined based on hardware real-time requirements and the time scale of microgrid operating condition changes. Considering the control cycle $T_{c}=50$ ms, updating the policy every 50 cycles not only matches the typical fluctuation time scale of load/photovoltaic output, but also ensures that the computational load of the STM32H7 controller is below 25%. That is, a single policy update takes less than or equal to 15 ms, and performing 10 iterations per update reduces the loss function by 95%; continuing to 20 iterations does not yield a significant performance improvement. Therefore, performing 10 iterations per update is the computationally optimal number.

Simulation scenario 1 is configured as follows: The total simulation time is 2.5 s, and the active power capacity of Agent 3 is set to half that of Agents 1 and 2. After system startup, only Load 1 (20 kW + 20 kVar) is connected to the circuit during the period from 0 s to 1 s, with no additional load disturbance. At t = 1 s, Load 2 is connected, increasing the total load to 30 kW + 30 kVar. Simultaneously, the grid-connected system capacity corresponding to Agent 3 is doubled, making its capacity equal to that of Agents 1 and 2 thereafter. This configuration is intended to validate the system’s dynamic response to step load increases and its power redistribution capability. At t = 2 s, Load 2 is disconnected, and the total load returns to 20 kW + 20 kVar. The real-time electricity price within the 2.5 s simulation period is set as follows: 0.2 CNY/kWh from 0 to 0.2 s, rising to 0.8 CNY/kWh from 0.5 to 1 s, and maintained at 0.3 CNY/kWh from 1 to 2.5 s.

**Figure 3:**
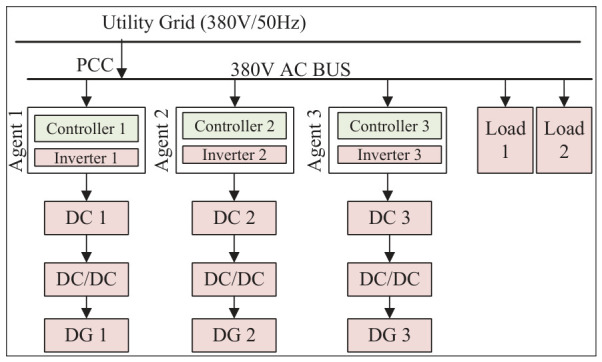
Physical connection topology diagram.

The physical connection topology diagram of each component in the microgrid is presented in Figure 3.

In Figure 3, Inverters 1/2/3 correspond to Agents 1/2/3 in the paper, respectively; DG 1 and DG 2 are photovoltaic units, and DG 3 is an energy storage unit. The rated DC bus voltage is $U_{dc}=700V$; the rated AC bus voltage is 380 V / 50 Hz; Load 1: 20 kW + 20 kVar; Load 2: 10 kW + 10 kVar.

**Figure 4:**
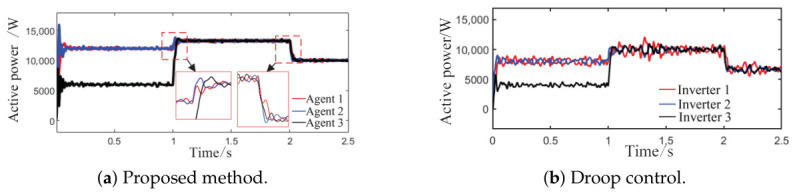
Active power sharing among agents under Scenario 1 (load step change).

**Figure 5:**
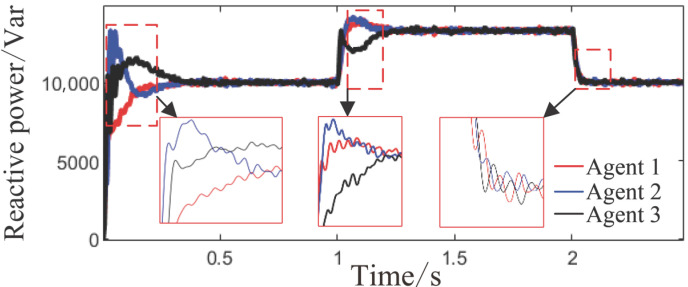
Reactive power sharing among agents under the proposed method.

As observed in Figure 4a,b and Figure 5, when the load or the capacity of the grid-connected inverter system changes, active and reactive power are successfully redistributed among the agents. This demonstrates that the proposed method comprehensively considers economic efficiency, stability, safety, and real-time electricity price, with the multi-agent adaptive decision network dynamically allocating power. Inverters with larger capacities bear a greater share of the power; during peak electricity price periods, photovoltaic inverters with lower generation costs are prioritized to increase output, thereby improving economic efficiency; when an inverter approaches its power limit, its output is constrained by the barrier Lyapunov function.

**Figure 6:**
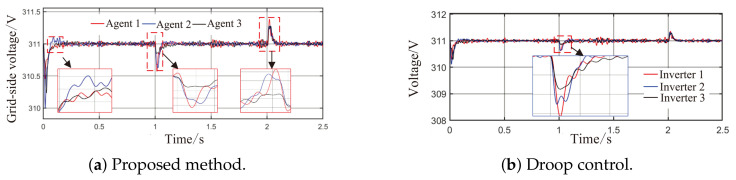
Grid-side voltage waveforms under Scenario 1.

**Figure 7:**
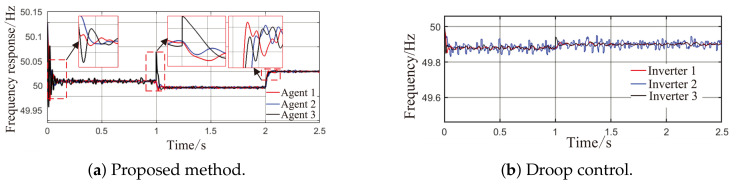
Frequency response waveforms under Scenario 1.

From Figure 6a and Figure 7b, it can be seen that the proposed method rapidly eliminates voltage deviations caused by load variations. Under the most extreme conditions, the voltage fluctuation is only 1.6 V, and it quickly recovers to stability within 0.01 s. The frequency remains consistently near 50 Hz. These results indicate that the proposed method effectively eliminates voltage or frequency errors induced by load or capacity changes.

**Figure 8:**
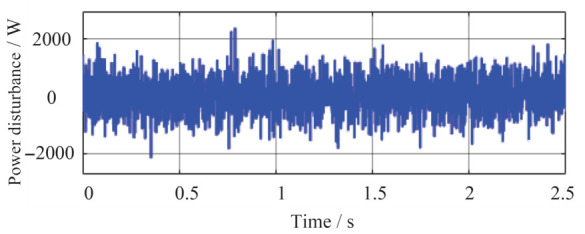
Active power disturbance diagram.

Simulation scenario 2 is configured as follows. The total simulation time is 2.5 s, and the active power capacity of Agent 3 is set to half that of Agents 1 and 2. After system startup, both Load 1 and Load 2 are connected to the circuit throughout the period from 0 to 2.5 s. At t = 1 s, the grid-connected system capacity corresponding to Agent 3 is doubled, making its capacity equal to that of Agents 1 and 2 thereafter. The load disturbance configuration is shown in Figure 8, aiming to validate the system’s dynamic response to load disturbances and its power redistribution capability.

**Figure 9:**
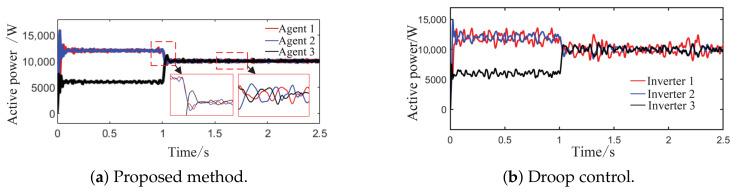
Active power sharing among agents under Scenario 2 (continuous load disturbance).

**Figure 10:**
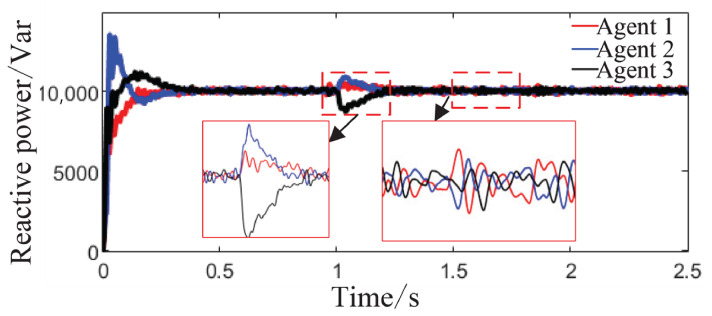
Reactive power sharing among agents under the proposed method.

**Figure 11:**
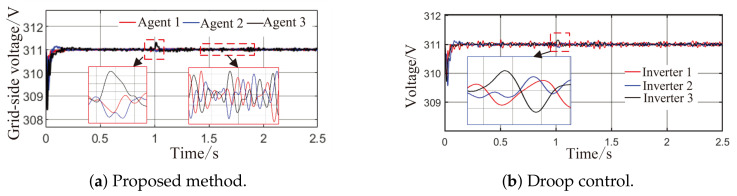
Grid-side voltage waveforms under Scenario 2.

**Figure 12:**
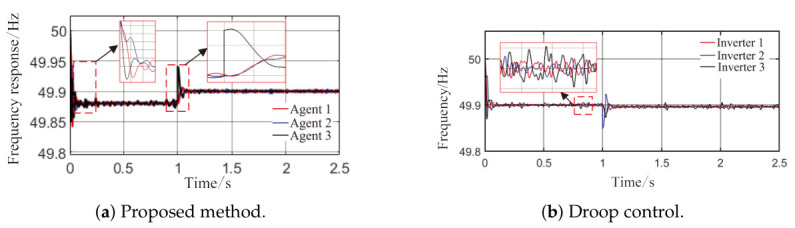
Frequency response waveforms under Scenario 2.

As shown in Figure 9a, Figure 10, Figure 11a and Figure 12a, in the presence of load disturbances, active and reactive power are successfully redistributed among the agents. The proposed method rapidly eliminates voltage or frequency deviations caused by load or capacity changes, further confirming its effectiveness.

Comparing the waveforms of the droop control method and the proposed method reveals that under step load changes and continuous random power disturbances, conventional droop control exhibits notable overshoot and oscillation, with a maximum voltage fluctuation of 2.8 V and a maximum power fluctuation of 2 kW. In contrast, the proposed method achieves smaller voltage and power fluctuations than those of droop control. Regarding the shape of the curves, the output power trajectories of the distributed generation units in the proposed method are highly consistent, enabling rapid and balanced power redistribution after load changes without obvious power sharing lag. Compared with conventional droop control, the proposed method resolves the problem of fixed parameters being unable to adapt to dynamic operating conditions through topology awareness and adaptive decision-making, while also preventing voltage and frequency violations via a safety constraint module.

Furthermore, the total generation cost per unit time, denoted as $C_{\mathrm{total}}$, is adopted to evaluate the economic performance in this paper. The specific calculation method is as follows:

(48) $$C_{\mathrm{total}}=C_{\mathrm{grid}}+C_{\mathrm{loss}}+C_{\mathrm{pv}}$$

where $C_{\mathrm{grid}}$ is the cost of electricity purchased from the main grid, calculated based on the real-time electricity price curve from the simulation; $C_{\mathrm{loss}}$ denotes the cost of charging and discharging losses of the energy storage system, converted at a charge/discharge efficiency of 90%; and $C_{\mathrm{pv}}$ represents the operation and maintenance cost of photovoltaic generation, taken as 0.05 CNY/kWh. The average total operating cost of the proposed method is 12.76 CNY, while that of the conventional droop control method is 13.90 CNY, representing a relative improvement of 8.2%.

**Figure 13:**
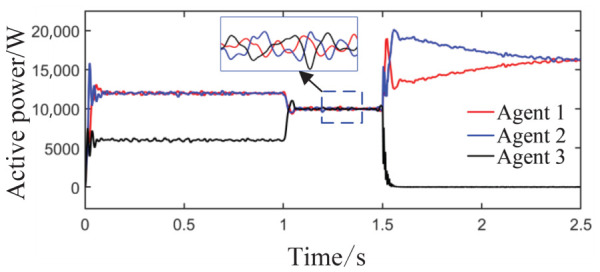
Active power sharing among agents.

**Figure 14:**
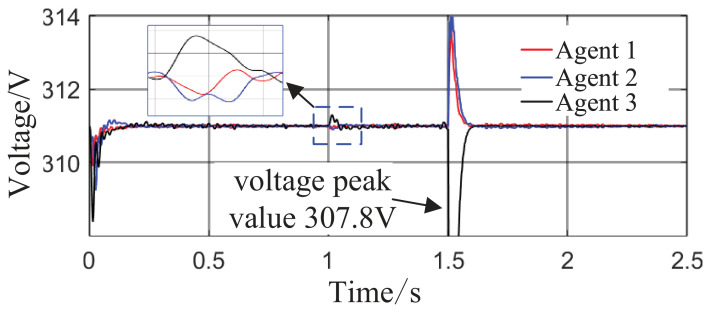
Grid-side voltage waveform.

**Figure 15:**
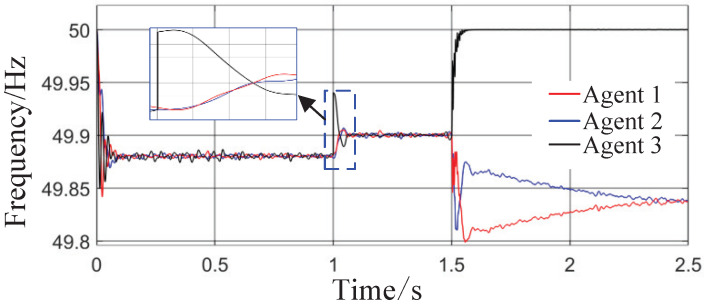
Frequency response waveform.

Scenario 3: The total simulation time is 2.5 s. The active power capacity of Agent 3 is set to half that of Agents 1 and 2. After system startup, both Load 1 (20 kW + 20 kVar) and Load 2 (10 kW + 10 kVar) are always connected to the circuit during the period from 0 s to 2.5 s. At t = 1 s, the active capacity of Agent 3 is doubled. At t = 1.5 s, the values of $k_{V}$, $k_{f}$, and $k_{P}$ are abruptly changed from their original values of 0.8, 1, and 0.6 to 0.6, 1.2, and 0.8, respectively; meanwhile, Agent 3 is directly disconnected from the circuit. This configuration is intended to test the stability of control performance under sudden changes in key parameters or agent failure. The active power sharing among the three agents is shown in Figure 13, and the grid-side voltage and frequency waveforms are shown in Figure 14 and Figure 15, respectively.

As observed in Figure 13, during the initial stage from 0 to 1 s, the power sharing ratio matches well with the capacities of the units. After the capacity of Agent 3 is doubled at t = 1 s, the system accomplishes power redistribution without noticeable oscillation, and the three agents quickly converge to an equal output state. At t = 1.5 s, when the three decay coefficients of the barrier Lyapunov function simultaneously change and Agent 3 is disconnected as an extreme fault, the remaining two agents achieve global power balance within approximately 0.2 s, eventually stabilizing at an equal output of 15 kW each within 1 s. Although a brief power overshoot of about 18 kW occurs at the moment of the fault, no sustained oscillation is observed, demonstrating the fault-tolerant advantage of the distributed multi-agent architecture without the need for central node intervention.

From Figure 14 and Figure 15, it can be seen that during the period from 0 to 1 s, the system voltage is stable at approximately 311 V, and the frequency remains around 49.88 Hz. When the capacity of Agent 3 changes at t = 1 s, a voltage fluctuation with an amplitude of 0.3 V occurs, which quickly recovers to stability within 0.1 s. When the disturbance occurs at t = 1.5 s, the voltage exhibits an instantaneous peak of 313.4 V and a nadir of 307.8 V, with a total fluctuation amplitude of 3.2 V, but the voltage rapidly returns to a stable value within 0.2 s. The frequency always remains between 49.8 and 50 Hz, without significant fluctuations throughout the process. Thus, even under extreme operating conditions, the operational indices of the microgrid strictly satisfy safety operation standards. This indicates that the safety constraint mechanism based on the barrier Lyapunov function possesses strong parameter robustness: even when the control parameters undergo large abrupt changes, it effectively mitigates the risk of transient violations. Moreover, the adaptive decision network can adjust the control strategy online, quickly counteracting the effects of parameter drift and topology mutation.

The experimental results of Scenario 3 further validate the adaptability and reliability of the proposed method under complex and variable operating conditions. The method not only exhibits excellent dynamic performance and economic efficiency under normal conditions, but also ensures safe and stable system operation under extreme scenarios such as abrupt key parameter changes and sudden agent withdrawal.

**Figure 16:**
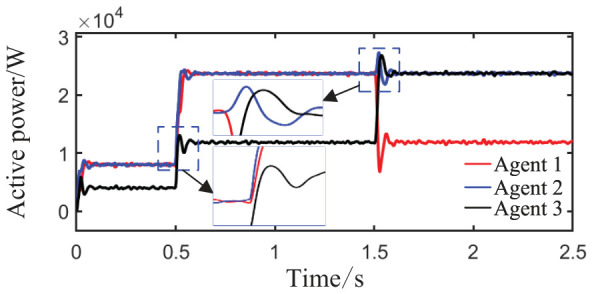
Active power sharing among agents.

**Figure 17:**
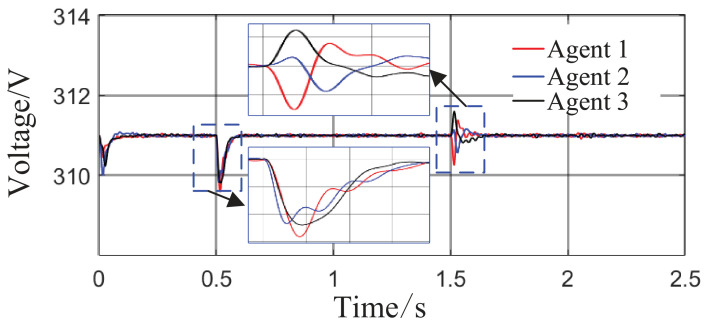
Grid-side voltage waveform.

**Figure 18:**
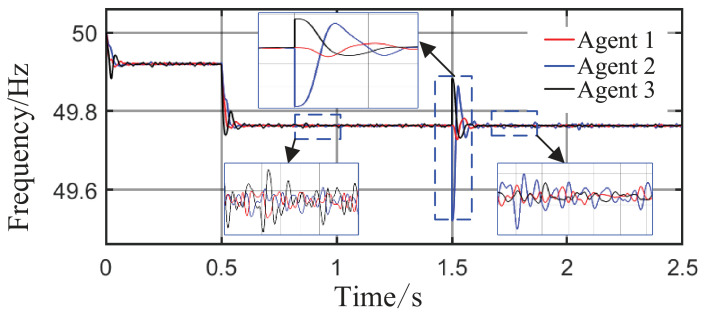
Frequency response waveform.

Scenario 4: The total simulation time is 2.5 s. The active power capacity of Agent 3 is set to half that of Agents 1 and 2. After system startup, only Load 1 (20 kW + 20 kVar) is connected during the period from 0 to 0.5 s. At t = 0.5 s, Load 3 (40 kW + 40 kVar) is connected to simulate the impact of the maximum load surge on the microgrid. At t = 1.5 s, the capacity of Agent 3 is doubled, while the capacity of Agent 2 is halved, to test the control performance under drastic topology changes. The active power sharing among the three agents is shown in Figure 16, and the grid-side voltage and frequency waveforms are shown in Figure 17 and Figure 18, respectively.

As shown in Figure 16, during the initial stage from 0 to 0.5 s, the power sharing ratio matches well with the capacities of the units. At t = 0.5 s, when a load surge of 40 kW + 40 kVar is applied, the total load suddenly triples, yet the system achieves power redistribution within approximately 0.1 s without sustained oscillation, maintaining a 2:2:1 output ratio that matches the unit capacities. At t = 1.5 s, when the topology undergoes drastic changes (the capacity of Agent 3 doubles while that of Agent 2 halves), the system rapidly adjusts its output strategy within 0.25 s, finally converging to a 1:1:2 output ratio that fully matches the new capacity proportion. Although a brief power overshoot of about 25 kW occurs in Agent 1, no power violation or system instability is observed.

From Figure 17 and Figure 18, in the steady-state stage from 0 to 0.5 s, the system voltage is stable at 311 V, the frequency remains at 49.9 Hz, and the fluctuations are within 0.3 V and 0.02 Hz, respectively. At t = 0.5 s, when the maximum load surge occurs, the voltage drops to a minimum of 309.8 V, and the frequency reaches a minimum of 49.78 Hz, but both recover to steady-state values within 0.05 s. At t = 1.5 s, under the drastic topology change, the voltage fluctuation amplitude is controlled within 1.6 V, and the frequency drops only to a minimum of 49.52 Hz, quickly recovering to above 49.8 Hz within 0.018 s. All operational indices consistently satisfy grid-connection requirements, demonstrating that the safety constraint mechanism based on the barrier Lyapunov function effectively avoids safety boundary violations under boundary operating conditions.

The experimental results of Scenario 4 further demonstrate the engineering practicability of the proposed method. Under extreme scenarios such as load surges and abrupt agent capacity changes, the method still achieves fast global power coordination and safe and stable operation, providing effective technical support for the reliable control of high-proportion renewable energy microgrids in complex and variable operating environments.

**Figure 19:**
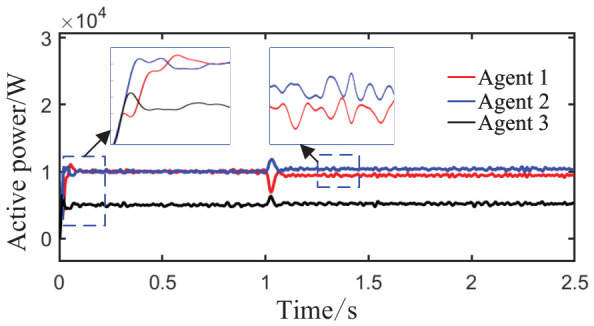
Active power sharing among agents of System 1.

**Figure 20:**
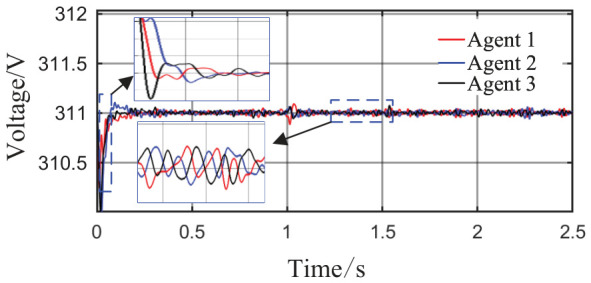
Grid-side voltage waveform of System 1.

**Figure 21:**
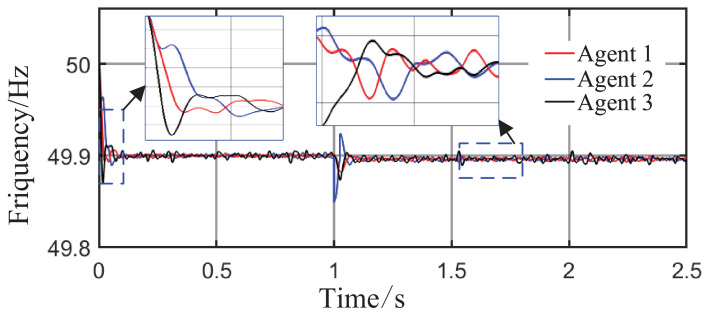
Frequency response waveform of System 1.

**Figure 22:**
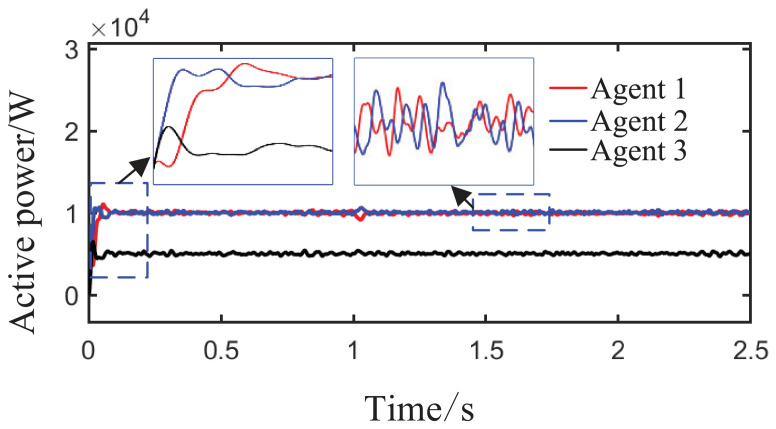
Active power sharing among agents of System 2.

**Figure 23:**
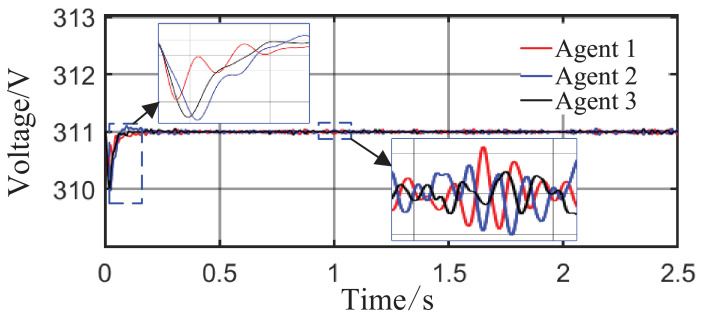
Grid-side voltage waveform of System 2.

**Figure 24:**
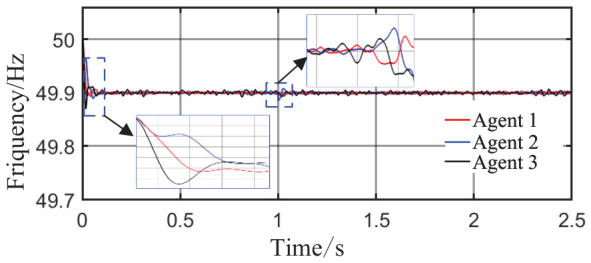
Frequency response waveform of System 2.

Scenario 5: The total simulation time is 2.5 s. Agents 1 and 2 have a capacity of 20 kW; Agent 3 has a capacity of 10 kW, and the load is always 25 kW + 25 kVar. At t = 1 s, the communication link between Agent 1 and Agent 3 is disconnected, and the actual topology becomes a 1-2-3 chain structure. Two systems are configured: System 1 adopts the complete method proposed in this paper; System 2 fixes the G matrix as the initial fully connected matrix without updating it according to the communication status. The active power sharing among the three agents in System 1 is shown in Figure 19, and the grid-side voltage and frequency waveforms are shown in Figure 20 and Figure 21, respectively. The active power sharing among the three agents in System 2 is shown in Figure 22, and its grid-side voltage and frequency waveforms are shown in Figure 23 and Figure 24, respectively.

Comparing Figure 19 and Figure 22, before the communication link disconnection at t = 1 s, both systems achieve accurate active power sharing in proportion to their capacities (2:2:1). After the communication link disconnection, the power curves of System 1 exhibit very small fluctuations, and the power sharing ratio remains stable. This is because the topology identification module quickly detects the link interruption and automatically updates the topology correlation matrix G by setting $g_{13}$ and $g_{31}$ to 0, so that Agent 1 communicates only with Agent 2, and Agent 3 only with Agent 2. Global power coordination is achieved through the relay function of Agent 2. In contrast, because System 2 fixes the topology matrix as the initial fully connected state, it still attempts to send coordination information to the non-existent link, preventing direct exchange of decision data between Agent 1 and Agent 3. At t = 1 s, a transient power oscillation with an amplitude of approximately 2 kW occurs, and the system only returns to steady state after an adjustment period of about 0.1 s, but the power sharing ratio does not strictly follow the 2:2:1 proportion.

Comparing Figure 20 and Figure 23, after the communication link disconnection, the grid-side voltage of System 1 remains stable at 311 V without noticeable fluctuations. In contrast, System 2 exhibits a voltage spike with an amplitude of approximately 0.1 V at t = 1 s.

Comparing Figure 21 and Figure 24, the frequency of System 1 always remains around 49.9 Hz, with almost no impact from the communication link disconnection. However, System 2 experiences a significant frequency drop at t = 1 s, with a minimum value of approximately 49.85 Hz, returning to steady state after about 0.1 s of adjustment.

**Figure 25:**
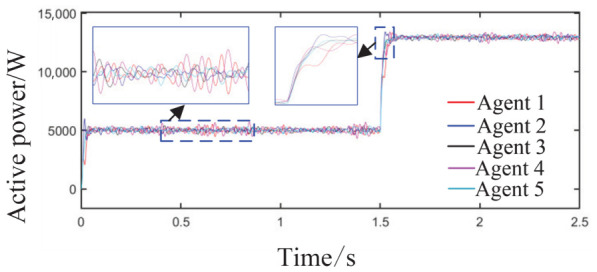
Active power sharing among agents.

**Figure 26:**
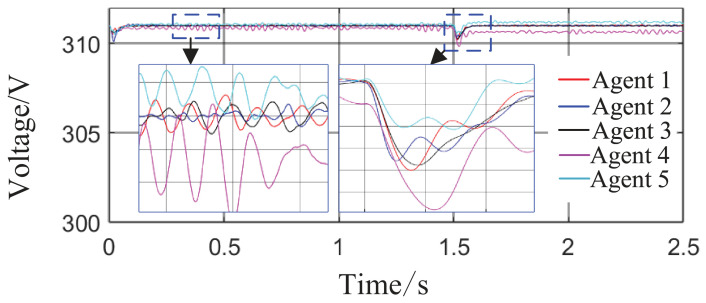
Grid-side voltage waveform.

**Figure 27:**
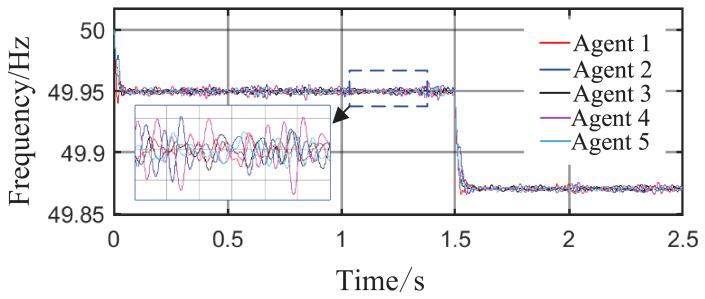
Frequency response waveforms under Scenario 6.

Scenario 6: The total simulation time is 2.5 s. After system startup, during the period from 0 s to 1 s, a load of 25 kW + 25 kVar is connected to the circuit, with no additional load disturbance. At t = 1 s, Load 2 is connected, increasing the total load to 65 kW + 65 kVar. The active power sharing among the five inverters is shown in Figure 25, and the grid-side voltage and frequency waveforms are shown in Figure 26 and Figure 27, respectively. Due to the time constraints of this revision, the computational limitations of the simulation platform, and the difficulty of modeling large-scale microgrid systems, we are currently unable to provide a complete large-scale simulation validation at the hundred-node level, for which we sincerely apologize. Our future work will focus on simulations of large-scale microgrid clusters with more than 50 nodes, as well as hardware-in-the-loop experiments, to further validate the scalability and robustness of the algorithm in practical engineering scenarios.

As shown in Figure 25, Figure 26 and Figure 27, from 0 to 1.5 s, the system operates in a stable grid-connected state, with each agent achieving precise and smooth active power sharing, with fluctuations kept within a small range and high steady-state sharing accuracy. The output voltage amplitude of each agent remains stable within the rated range, with controllable dynamic oscillation amplitude. The grid-side frequency remains stable near the rated power frequency, with minimal steady-state fluctuations. At the load change disturbance moment t = 1.5 s, a large power load switching disturbance occurs. Active power is redistributed instantaneously, and each agent responds quickly and adaptively adjusts its output active power. The terminal voltage experiences a short-term dynamic dip, but under the proposed distributed safety coordination control, the voltage quickly recovers and rises back to the rated operating range without persistent violations or instability.

## 6. Conclusions

To address the control challenges posed by high-proportion renewable energy integration in microgrids—namely strong power generation fluctuations, complex and variable loads, and dynamic topology reconfiguration—this paper proposes a distributed generation optimization control method based on multi-agent adaptive decision-making. The proposed method overcomes the shortcomings of conventional distributed control, such as insufficient global state awareness, lack of safety constraints, and poor dynamic adaptability. The core contributions and research conclusions are summarized as follows:

- To resolve the problem of incomplete decision-making information caused by static topology modeling and inefficient multi-source information fusion in traditional methods, a topology identification algorithm based on an improved Bayesian network is proposed. The physical distance and power interaction intensity are quantified into a dynamic topology correlation matrix, enabling real-time perception of the microgrid topology. A full-dimensional state vector encompassing equipment operating status, topological correlations, real-time electricity prices, and short-term load forecasts is dynamically weighted, solving the issues of information overload and decision bias, and providing a more refined input feature for the multi-agent decision-making system.
- To address the difficulty of conventional distributed control in balancing global optimization and operational safety, as well as its excessive reliance on a central node, a consensus protocol and a barrier Lyapunov function are integrated to design a fully distributed safety coordination module. This module achieves global optimal power allocation solely through local information exchange among neighboring agents. Without any central node intervention, it ensures that the control actions simultaneously satisfy both global economy and system operation safety, avoiding safety risks such as voltage violations and frequency instability commonly encountered in traditional methods.
- To overcome the limitation of existing multi-agent microgrid control methods that rely on offline training and cannot adapt to dynamic operating environments, an experience screening mechanism based on information entropy is proposed, retaining only high-information-gain experience data. Dynamic clipping parameters and an adaptive learning rate are introduced to achieve online dynamic adjustment of the decision network structure and activation function parameters, enabling agents to continuously and autonomously evolve during operation and maintain optimal decision-making performance. This solves the problem of severe performance degradation of offline-trained models in scenarios such as topology reconfiguration.

Future research will focus on the coordinated optimization of multi-microgrid clusters to address scheduling and power balance challenges among large-scale distributed generation units. Implementing a distributed framework based on multi-agent deep reinforcement learning will enable dynamic energy trading and autonomous operational strategies improving system stability and economy. Furthermore simulation and experimental results prove that the proposed strategy rapidly generates practical multi-objective decisions under complex scenarios such as load fluctuations and topology reconfiguration. This significantly enhances the adaptability and operational economy of the microgrid, thereby contributing to its improved stability and flexibility. 

## Figures and Tables

**Table 1:** Parameter meaning.

Parameters	Symbol Meaning
P,Q,V	active power, reactive power, RMS voltage at the PCC
*f*	system frequency
p(t),q(t)	instantaneous active power, instantaneous reactive power
$v_{\mathrm{PCC}}$	instantaneous voltage at the PCC
$v_{d},v_{q}$	d-q axis voltages
$i_{d},i_{q}$	d-q axis currents
$v_{a},v_{b},v_{c}$	three-phase instantaneous voltages
$i_{a},i_{b},i_{c}$	three-phase instantaneous currents
$P_{L}$	load forecast value
$\Delta P_{\mathrm{req}}$	total active power regulation demand
$P_{c}$	current operating power
$P_{\mathrm{ref}}$	command power value
$U_{\mathrm{dc}}$	DC bus voltage
*G*	topology correlation matrix
$g_{ij}$	the *i*-th row and j-th column of topology correlation matrix
$d_{ij}$	physical distance between the i-th and j-th distributed generation units
$P_{\mathrm{int},ij}$	power interaction between the i-th and k-th distributed generation units
$P_{\mathrm{int},ik}$	power interaction between the i-th and k-th units
α	distance decay coefficient
β	power weighting coefficient
*N*	the total number of distributed generation units within the microgrid
*O*	state observation vector
$O_{w}$	weighted state observation vector
*W*	attention weight matrix
$w_{k}$	attention weights
σ(·)	attention weights for the corresponding state dimensions
$\alpha_{d}$	attenuation coefficient
$V_{\max},V_{\min}$	maximum and minimum allowable voltage
$P_{\max},P_{\min}$	maximum and minimum allowable active power
$f_{\max},f_{\min}$	maximum and minimum allowable frequency
$B_{V},B_{f},B_{P}$	voltage safety barrier function, frequency safety barrier function, power safety barrier function
$k_{V},k_{f},k_{P}$	decay coefficients of voltage, frequency and power
$k_{\mathrm{PV}},k_{\mathrm{PF}}$	power–voltage coupling coefficient, power–frequency coupling coefficient
$e_{c}$	global coordination error
$\Delta P_{r}$	maximum allowable power change rate
$k_{p},k_{r}$	proportional coefficient, resonant coefficient
$v_{ff},i_{ff}$	grid voltage feedforward, load disturbance feedforward
$Z_{\mathrm{eq}}$	equivalent system impedance
$v_{d,\mathrm{ref}},v_{q,\mathrm{ref}}$	d-q axis voltage references
θ	phase angle of the reference voltage vector
$a_{1}$	preliminary control action
$a_{2}$	final control action
$\Delta P_{1},\Delta Q_{1}$	active power adjustment, reactive power adjustment
$\Delta P_{2}$	final active power adjustment
$\Delta P_{2}^{\prime }$	candidate final active power adjustment
*L*	multi-objective loss function
$L_{e},L_{s},L_{\mathrm{sf}}$	economic loss, stability loss, safety loss
$w_{1},w_{2},w_{3}$	dynamic weight coefficients
*r*	reward value
$r_{e},r_{s},r_{\mathrm{sf}}$	economic reward, stability reward, safety reward
$G_{\mathrm{info}}$	information gain
$\gamma^{\prime }$	discount factor
$\lambda_{\mathrm{gae}}$	GAE parameter
ι(t)	dynamic clipping parameter
η(t)	adaptive learning rate
ξ	experience screening unit collects experience data
$\delta_{t}$	temporal-difference error
$\hat{A}$	advantage function estimate value
φ	parameter set
$V_{\varphi }\left(\cdot \right)$	value network
$V_{\varphi^{\prime }}\left(\cdot \right)$	target value network
$E_{\xi ∼D}\left[\cdot \right]$	expectation over the data in the buffer
r(φ)	policy probability ratio
$\pi_{\varphi }$	probability density
H(·)	entropy
λ	real-time electricity price
$\lambda_{\mathrm{avg}}$	average electricity price over the past hour
$\lambda_{\mathrm{peak}}$	peak electricity price of the day
$P_{\mathrm{avg}}$	average power over the past hour

**Table 2:** Simulation model parameter.

Parameters	Values
control cycle $T_{c}$	10 ms
switching cycle $T_{s}$	100 μs
$U_{\mathrm{dc}}$	700 V
$V_{\mathrm{nom}}$	380 V
α	0.01
β	0.8
*N*	3
safe operating voltage range	361∼399 V
safe operating frequency range	49.5∼50.5 Hz
$k_{V},k_{f},k_{P}$	0.8, 1, 0.6
$k_{p},k_{r}$	0.8, 20
$k_{\mathrm{PV}},k_{\mathrm{PF}}$	0.02, 0.01
$\gamma^{\prime }$	0.985
$\lambda_{\mathrm{gae}}$	0.95
$\tau \left(0\right)$	0.2
η(0)	0.001
Load 1	20 kW + 20 kVar
Load 2	10 kW + 10 kVar
power disturbance amplitude (simulation scenario 2)	±2000 W
$Z_{\mathrm{eq}}$	0.5+j0.3 Ω

## Data Availability

Data are contained within the article.

## Abbreviations

The following abbreviations are used in this manuscript:

RMS	Root mean square
PCC	Point of common coupling
PWM	Pulse-width modulation
CAN	Controller Area Network
ELU	Exponential Linear Unit
PPO	Proximal policy optimization
